# Evaluation of the Bonebridge BCI 602 active bone conductive implant in adults: efficacy and stability of audiological, surgical, and functional outcomes

**DOI:** 10.1007/s00405-022-07265-2

**Published:** 2022-02-19

**Authors:** Katarzyna B. Cywka, Piotr H. Skarzynski, Bartlomiej Krol, Stavros Hatzopoulos, Henryk Skarzynski

**Affiliations:** 1 Otorhinolaryngosurgery Clinic, World Hearing Center, Institute of Physiology and Pathology of Hearing, Warsaw/Kajetany, Poland; 2 Teleaudiology and Screening Department, World Hearing Center, Institute of Physiology and Pathology of Hearing, Mokra 17 Street, Nadarzyn, 05-830 Warsaw/Kajetany, Poland; 3 Heart Failure and Cardiac Rehabilitation Department, Faculty of Medicine, Medical University of Warsaw, Warsaw, Poland; 4 Institute of Sensory Organs, Kajetany/Warsaw, Poland; 5 Department of Audiology and ENT, University of Ferrara, Ferrara, Italy

**Keywords:** Bone conduction, Bonebridge, Bone conduction implant, Hearing loss, Transcutaneous hearing implant, Quality of life, Partial deafness treatment

## Abstract

**Purpose:**

(1) To assess the effectiveness and safety of a bone-conduction implant, the Bonebridge BCI 602, in adults with conductive or mixed hearing loss. (2) To investigate whether the Bonebridge BCI 602 is at least as effective as the Bonebridge BCI 601 in such patients.

**Methods:**

The study group included 42 adults who had either conductive or mixed hearing loss. All patients underwent Bonebridge BCI 602 implant surgery. Before and after implantation, pure-tone audiometry, speech recognition tests (in quiet and noise), and free-field audiometry were performed. Word recognition scores were evaluated using the Polish Monosyllabic Word Test. Speech reception thresholds in noise were assessed using the Polish Sentence Matrix Test. Subjective assessment of benefits was done using the APHAB (Abbreviated Profile of Hearing Aid Benefit) questionnaire.

**Results:**

The APHAB questionnaire showed that difficulties in hearing decreased after BCI 602 implantation. Both word recognition in quiet and speech reception threshold in noise were significantly better after BCI 602 implantation and remained stable for at least 12 months. A significant advantage of the device is a reduced time for surgery while maintaining safety. In this study, the mean time for BCI 602 implantation was 28.3 min ± 9.4.

**Conclusions:**

The second-generation Bonebridge BCI 602 implant is an effective hearing rehabilitation device for patients with conductive or mixed hearing loss. Patient satisfaction and audiological results confirm its efficacy and safety. Its new shape and dimensions allow it to be used in patients previously excluded due to insufficient or difficult anatomical conditions. The new BCI 602 implant is as effective as its predecessor, the BCI 601.

## Introduction

There are an increasing number of rehabilitation and treatment options for patients with conductive or mixed hearing loss, and professionals need to be able to inform their patients about their options based on actual experiences and results. Currently, the most common treatments for conductive and mixed hearing loss are drugs, middle ear surgery, hearing aids, bone-conduction hearing implants, or a combination of these [1, 2]. The most common disorders described in the scientific literature are abnormality or malformation of the ossicular chain, otosclerosis, chronic ear disease, and cholesteatoma. Most interventions to treat these conditions are a type of tympanoplasty or bone-conduction implant.

A large group of patients are patients with congenital anatomical defects of the outer and/or middle ear who cannot use the traditional methods of treatment and whose surgical procedures have not given satisfactory results. A possible way to improve their hearing is to use implantable or non-implantable bone-conduction devices [3, 4]. For adults with conductive or mixed hearing loss, there are a number of devices on the market that are based on bone-conduction amplification. The devices differ both visually and functionally. An important aspect is the fitting range of the device and the method of placement.

Before surgery, a patient must undergo a diagnostic process that qualifies them for the use of a specific device. This involves a consultation with an audiologist, hearing care professional, neurologist, psychologist, radiologist, and speech therapist [5, 6]. Before and after any proposed intervention—such as middle ear surgery, implantation of a bone-conduction hearing device, or middle ear implants [4]—measurements are needed of hearing performance, and this involves testing hearing thresholds (pure-tone audiometry, over headphones, and in free field), speech tests in quiet and noise, and questionnaires. Before the final choice, a simulation of the use of a bone-conduction hearing aid is needed, making it possible to assess the real benefits for both the specialist and the patient. The patient should know the possibilities and limitations of a particular solution and know how the implant looks and works.

Often, the use of an implantable device is limited by insufficient or difficult anatomical conditions. Solutions using bone-conduction amplification are constantly improving, and this applies to both the external part (responsible for the reception, transmission, sound quality, and which can sometimes be adapted to the needs of the individual patient) and the internal part, which vibrates the bone of the skull, thus transmitting sound directly to the inner ear. One solution for patients with conductive or mixed hearing loss is the Bonebridge (BB) bone-conduction implant (Med-El, Innsbruck, Austria).

**Fig. 1:**
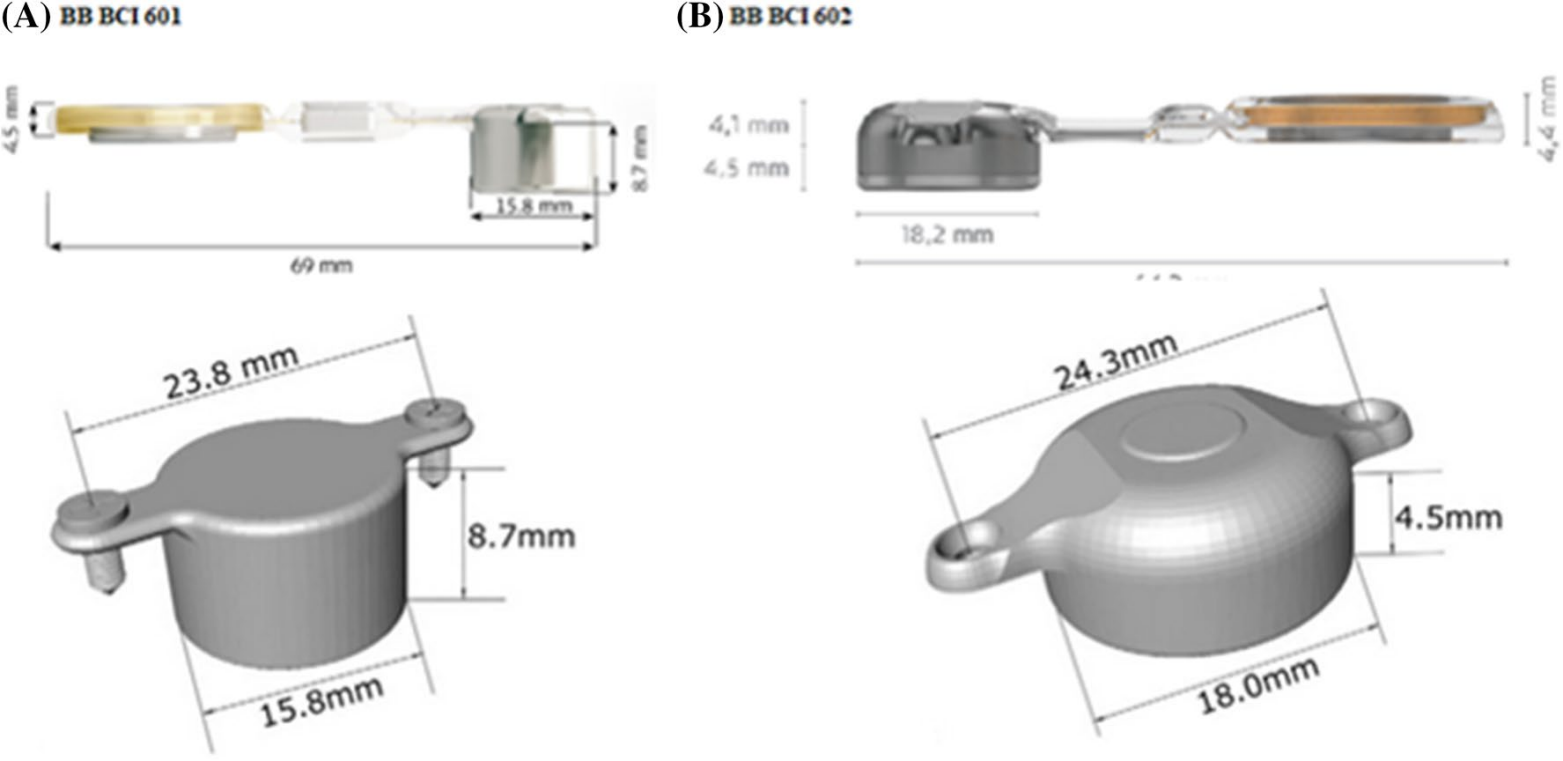
Two generations of Bonebridge implants. **A** Dimensions of the first-generation BCI 601. **B** Dimensions of the second-generation BCI 602

The Bonebridge was first implanted in 2011 as part of a clinical trial and was launched onto the EU market in September 2012 [7, 8]. The BC 601 consists of an external part (an audio processor) and an internal part—a bone-conduction ‘floating mass transducer’ (BC-FMT) that is surgically implanted into the skull in either the transmastoid, retrosigmoid, or middle fossa region. The second-generation Bonebridge implant, the BCI 602, has been available since 2019. It differs from its predecessor by the size of the internal BC-FMT. The upgrade gives the same power output for effective amplification, but requires nearly 50% less drilling depth. As illustrated in Fig. 1, the BCI602 is almost half the thickness of the previous generation [9, 10]. The external part—audio processor “Samba 1”—is the same for both devices. The audio processor is an advanced technological device that enables easier listening with automatic sound adaptation. Due to its dimensions: 30 × 35 × 10 mm and weight: < 9 g (including battery). The processor has 16-band digital equalizer, 16 independent compression channels, sound smoothing, speech and noise management, adaptive directional microphones, intelligent sound adapter, classifier 5 different programs, wind noise reduction, ambient sound, speech cracking, omnidirectional microphones, audio frequency range 250–8 kHz, wireless connectivity options via Bluetooth or telecoil to external devices such as mobile phones, and FM-systems. The processor is powered, by one non-rechargeable 675 zinc-air battery with, a nominal 1.4 Volt supply and 600 mA Hrs of capacity. Remote control allows the patient to adjust the volume and change programs depending on individual needs.

There are many studies in the literature showing the effectiveness of the BCI 601 implant, and the device can provide improved hearing in both children and adults [8, 11–18]. The primary aim of this study is to evaluate the effectiveness of the Bonebridge BCI 602 implant in a group of adult patients with conductive or mixed hearing losses. A second aim was to demonstrate that the Bonebridge BCI 602 implant is effective and safe in adult patients, and that the results with it are as good as with the previous BCI 601.

## Materials and methods

### Study subjects

The study group consisted of 42 adult patients, 19–74 years old (mean 40.5 years, SD = 14.8). There were 24 women and 19 men, and 23 patients had mixed and 19 had conductive hearing loss. In terms of ears, 23 were implanted on the right and 19 on the left. The causes of hearing loss were chronic otitis media (*n* = 14), chronic otitis media with cholesteatoma (*n* = 7), microtia and atresia (*n* = 13), atresia (*n* = 3), congenital defect of the middle ear (*n* = 2), congenital defect of the outer and middle ear (*n* = 1), middle ear tumor (*n* = 1), and tympanosclerosis (*n* = 1). The patients were selected according to the following criteria: patients had to be older than 18 years of age, ear reconstruction was completely finished, cooperative in audiological testing, and hearing thresholds had to accord with the manufacturer’s suggested criteria—conductive or mixed mild-to-moderate hearing loss; pure-tone average (PTA) BC threshold (measured at 0.5, 1, 2, 3, and 4 kHz) ≤ 45 dB HL, BC thresholds over the last 12 months were stable, and appropriate anatomical conditions had been confirmed by computed tomography. In addition, patients were required to have realistic expectations of the benefits and know the limitations of the Bonebridge BCI 602.

The operation side was selected after comparing hearing losses and anatomical conditions in the two ears. If there was a bilateral disorder, then the worse ear was selected. However, if there were no significant differences in hearing loss between both ears, in temporal bone structure, and a pre-operative hearing test showed that there were the same estimated benefits from a BC hearing aid, the patient could choose the side. In the diagnostic process, all patients were fitted with a reference device, a BC hearing aid on a soft band, and, based on in situ thresholds, the real and possible benefits of implantation were estimated. Before the operation, all patients underwent a CT scan of the temporal bone and a detailed assessment of anatomical conditions (analysis was carried out with the Otoplan device) [19, 20].

The study protocol was approved by the Institutional Review Board of the Institute of Physiology and Pathology of Hearing (IFPS:/KB/7/2020) and conformed with the Declaration of Helsinki. All patients signed an informed consent document prior to enrollment in the study.

### Statistical analysis

A Kolmogorov–Smirnov test was used to check whether the analyzed variables were normally distributed. A repeated-measures analysis of variance with Bonferroni adjustment for multiple comparisons was performed to test changes in mean scores over several time points. The *p* level was set at less than 0.05. All statistical tests were performed using IBM SPSS Statistics 24.

### Audiological assessment

All audiometric tests were performed before surgery as well as at activation and 6 and 12 months after surgery. Pure-tone thresholds were measured for air conduction (AC) and bone conduction (BC). Postoperatively, aided thresholds were measured using warble tones. Speech recognition in quiet was measured using the Demenko&Pruszewicz Polish Monosyllabic Word Test at 65 dB SPL presented via a loudspeaker. Speech reception thresholds in noise were assessed using the Polish Sentence Matrix Test [21]. The 50% speech reception threshold at a constant noise level of 65 dB SPL (SRT) was measured pre- and post-operatively with speech and noise presented from the front (S_0_N_0_) and the contralateral ear double-blocked with an earplug. The non-implanted ear was blocked but not masked, since masking would reduce the sensitivity of that ear to bone-conducted sound that is normally also heard from an implant on the other side of the skull. The Polish Sentence Matrix Test (PSMT) was used to measure intelligibility of speech presented against a background noise, and consists of five columns containing 10 names, 10 verbs, 10 numerals, 10 adjectives, and 10 nouns. For all patients, we simulated the result of a bone-conduction implant using a bone-conduction processor on a soft band (best-aided). Before surgery, we measured speech recognition (in quiet and noise) for the unaided and best-aided conditions, and made these same measurements after surgery using the recommended audio processor.

### Survey assessment

Patients’ satisfaction and quality of life were assessed before implantation and at 6 and 12 months post-operatively with the APHAB (Abbreviated Profile of Hearing Aid Benefit) questionnaire for adults. This questionnaire measures subjective hearing impairment on four different subscales pertaining to different listening situations and gauges what benefit the patient experiences after implantation [22]. Cox and Alexander [23] suggest that a change of at least 22 points on the subscales of Ease of Communication, Reverberation, and Background Noise indicates a clinically important change in speech intelligibility between aided and unaided conditions.

## Results

### Surgery

All implantations were carried out by two experienced surgeons as an in-patient procedure under general anesthesia. No complications occurred during surgery. To determine the optimal implant position, all patients underwent a CT scan of the temporal bone before the operation and a detailed assessment of anatomical conditions (with analysis carried out with the Otoplan device) [20, 24]. The surgery time ranged between 19 and 66 min (mean 28.3 min ± 9.4). The Bonebridge implant was placed in the previously made cavity in mastoid, and the plastic part bent through 0–90 degrees. The implant was attached using two custom screws (there was no need for lifts in either case). After checking the correct position of the implant, subcutaneous and skin sutures were applied (Polysorb 3.0 and Monosoft 2.0). After the procedure, all patients were in good condition and received antibiotics intravenously twice daily as well as pain medication. After 3 days in hospital, all subjects were discharged in good general condition, and received amoxicillin with clavulanic acid twice a day for 7 consecutive days with probiotic. Some 7–10 days after surgery, the dressings were removed as well as sutures from the retro auricular incision. There were no problems with wound healing, and patients reported no pain or other problems [8, 14]. From a surgical point of view, no cases were canceled due to mastoid size. For cases where there was no atresia/microtia, a superior–posterior C-shape cut was used (because the device tends to increase skin contour and elevate the skin; if a typical S-shape is used, there is a chance the skin at the anterior edge of the device could become thin and there might be risk of extrusion). In cases following pinna reconstruction, the cut should be performed in the line of the scar due to vascular conditions. An additional factor is the proper location of the device on the mastoid, which needs to be considered when choosing between the BCI 602 and BCI 601. Decision which device is better for each patient was taken based on CT scan with meticulous analysis of anatomical conditions. When the surface of the mastoid is more curved and there is the opportunity to adjust the implant, the authors recommend locating the device more superiorly, although it can be placed more temporally. With thickening of the bone, there might be a need to adjust the implant, pressing the dura a little through the thin bony plate. There is also the possibility of placing a thin layer of wax on the bottom. Wax can also be used in cases where there is contact through the aerated mastoid with the middle ear cavity, which prevents aeration of subcutaneous tissue from the tympanic cavity. In cases when there is risk of improper healing due to poor quality of the temporal bone surface, there is possibility to keep through 1–2 days Redon drainage with vacuum.

Four weeks (± 1 week) after the operation, the device was activated and the settings adjusted according to the audiometric test results, vibrogram results, and the patient's subjective assessment and comfort. All patients were provided with Samba 1(Med-El) audio processors (the first version of Samba), and the Symfit 7.0 program was used for fitting. All fittings were performed by two experienced audiologists. The strength of the sound processor’s magnet was adjusted to allow a stable hold while avoiding skin compression; in 22 cases, magnet No. 4 was used and in 20 cases magnet No. 3.

### Pure-tone audiometry

**Fig. 2:**
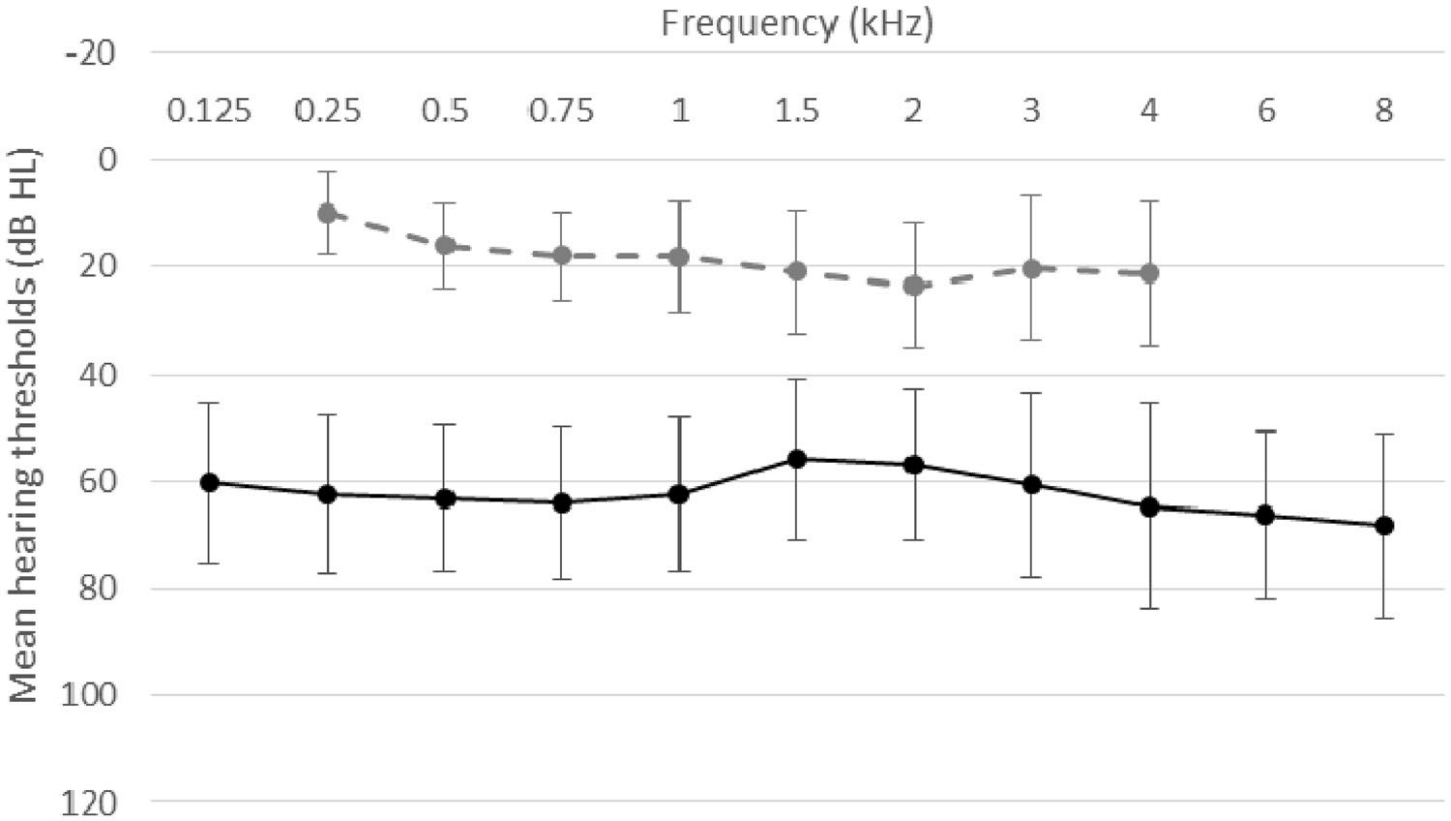
Thresholds for air conduction (black solid line) and bone conduction (gray dashed line). The bars are standard deviations

PTA (pure-tone average) for air conduction measured before implantation varied between 42.5 and 93.75 dB HL; *M* = 61.8, *SD* = 12.2. PTA for bone conduction varied between 2.5 and 35 dB HL; *M* = 19.7, *SD* = 7.1. Details for all frequencies are shown in Fig. 2.

### Functional gain

The mean functional gain estimated in the simulation before implantation was 28.8 dB HL (SD = 9.3). The real mean functional gain obtained 12 months after implantation was 27.0 dB HL (*SD* = 10.7).

### Sound field thresholds

**Fig. 3:**
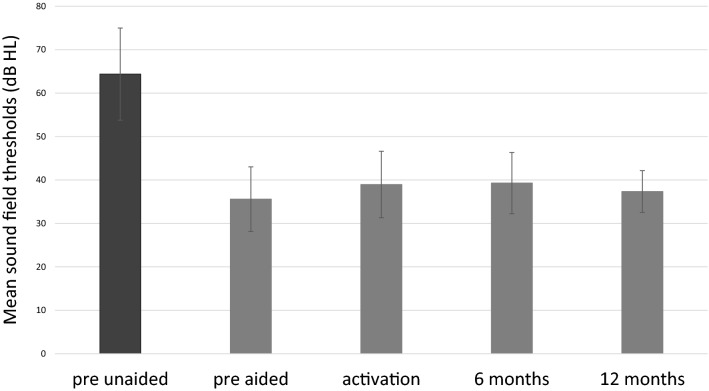
Sound field thresholds. All differences between pre-unaided and all other conditions are statistically significant at *p* < 0.001. The bars are mean scores; the error bars are standard deviations

The mean hearing thresholds in sound field before implantation were 64.4 dB HL (*SD* = 10.6), and in the simulation, they were found to be 35.6 dB HL (*SD* = 7.4). Statistically significant improvements were observed after surgery (*F* = 141.88; *p* < 0.001; *e*^2^ = 0.78). The mean hearing thresholds were 38.9 dB HL (*SD* = 7.6) at activation, 39.3 dB HL (*SD* = 7.1) after 6 months, and 37.4 dB HL (*SD* = 4.8) after 12 months (Fig. 3).

### Free-field word recognition in quiet

**Fig. 4:**
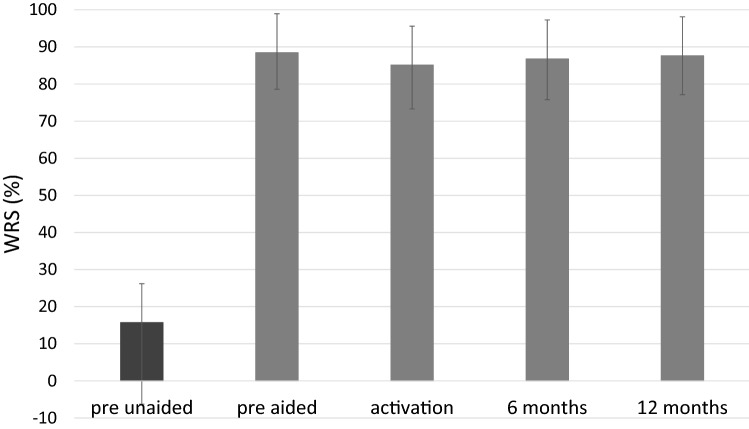
Word recognition scores (WRS) in quiet obtained in free-field audiometry at 65 dB SPL. All differences between pre-unaided and all other conditions are statistically significant at *p* < 0.001. The bars are mean scores; the error bars are standard deviations

Mean recognition scores (WRS) measured before implantation under headphones were 13.5% (*SD* = 22.8). Before implantation, they were measured unaided as 15.7% (*SD* = 22.0) and aided (in the simulation) as 88.5% (*SD* = 9.8). Statistically significant improvement was observed after surgery (*F* = 261.07; *p* < 0.001; *e*^2^ = 0.86). The mean WRS was 85.1% (*SD* = 11.8) at activation, 86.8% (*SD* = 11.0) after 6 months, and 87.6% (*SD* = 10.5) after 12 months (Fig. 4).

### Intelligibility of speech in noise

**Fig. 5:**
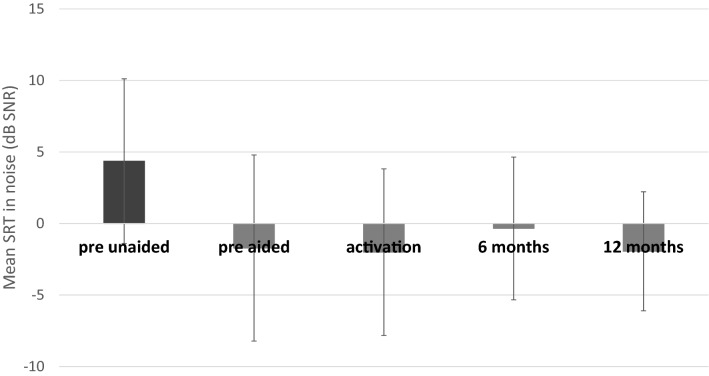
Speech reception thresholds (SRT) in noise. All differences between pre-unaided and all other conditions are statistically significant at *p* < 0.001. The bars are mean scores; the error bars are standard deviations

Mean speech reception thresholds (SRT) measured before implantation were unaided 4.36 dB SNR (*SD* = 5.76) and aided (in the simulation) − 1.72 dB SNR (*SD* = 6.51). Statistically significant improvement was observed after surgery (*F* = 17.14; *p* < 0.001; *e*^2^ = 0.30). The mean SRT was − 2.01 dB SNR (*SD* = 5.83) at activation, − 0.35 dB SNR (*SD* = 4.99) after 6 months, and − 1.95 dB SNR (*SD* = 4.16) after 12 months (Fig. 5).

### Difficulty in hearing (APHAB outcomes)

**Fig. 6:**
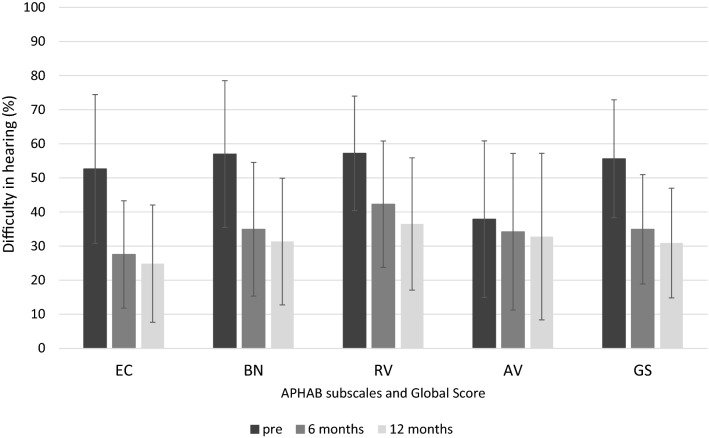
Results of APHAB questionnaire. *EC* Ease of Communication, *BN* Background Noise, *RV* Reverberation, *AV* Aversiveness, *GS* Global Score. The differences between pre and both follow-ups for the EC, BN, and RV subscales are statistically significant at *p* < 0.001; the difference for the AV subscale is statistically non-significant. The bars are mean scores; the error bars are standard deviations

The APHAB questionnaire showed that difficulties in hearing decreased after BB implantation (the lower the APHAB score, the better is the BB efficacy and patient satisfaction with hearing). There was a statistically significant improvement in the global score (*F* = 54.73; *p* < 0.001; *e*^2^ = 0.57) and the pre-operative score (*M* = 55.6; *SD* = 17.3) was significantly higher than both postoperative scores at 6 months (*M* = 34.9; *SD* = 16.0) and at 12 months (*M* = 30.9; *SD* = 16.1). Similarly, on the EC subscale (*F* = 55.90; *p* = 0.018; *e*^2^ = 0.58), the BN subscale (*F* = 37.29; *p* < 0.002; *e*^2^ = 0.48), and the RV subscale (*F* = 26.72; *p* < 0.002; *e*^2^ = 0.40), a significant change was found between pre-operative score and both 6 month and 12 month follow-ups. On the AV subscale, no difference was found between the three measurements (*F* = 1.73; *p* = 0.183). The mean APHAB outcomes are shown in Fig. 6.

Additionally, the amount of change in the individual APHAB outcomes was checked. A change of at least 22 points in the EC subscale was found in 45% of patients at the 6 month follow-up and in 52% of the patients at the 12 month follow-up. On the BN subscale, it was 43% and 50%, and in the RV subscale 36% and 43%, respectively.

## Discussion

The first-generation Bonebridge BCI 601 device was implanted in 2012. Since then, there have been many papers presenting the results of using the implant in children and adults. Audiological studies and questionnaires, as well as the different courses of surgery, have been analyzed. Numerous studies have shown the effectiveness of the BCI 601 in patients with conductive or mixed hearing loss or with single-sided deafness [1, 7–16, 20, 24]. In 2019, the second-generation BCI 602 implant was introduced. The main difference between the devices is the size of the internal part, the BC-FMT. The BCI 602 is almost half the thickness of the previous generation, allowing implantation with a drilling depth of only 4.5 mm [9, 10, 14, 25]. The reduced size allows the new device to be used in a wider group of patients. Patients who were previously excluded due to insufficient conditions for use of the BCI 601 can today undergo surgery using the BCI 602. The BCI 602 can be effectively applied in patients after a radical cavity operation or even after mastoid obliteration [26, 27]. It can be applied more effectively and there is a better chance of long-term results [28].

The first results of implantation of the BCI 602 were published in 2021 [25], with the observations and analysis based on 16 patients some 3 months after surgery. The authors obtained very good results: the mean functional gain (FG) at activation (2 weeks after surgery) in the MHL/CHL cohort was 25.9 ± 6.7 dB and increased significantly to 37.75 ± 4.40 dB after 3 months. The WRS (%) in quiet at activation and 3 months post-surgery significantly improved to 74.3 ± 18.0% and 85.6 ± 17.8%, respectively. The mean SRT (including various SNRs) improved significantly at the 3 month follow-up (from 56.2 ± 10.9 dB to 45.3 ± 10.5 dB and from 0.10 ± 3.4 dB to − 3.3 ± 2.5 dB, respectively). Our work included 42 adult patients and the follow-up was at 12 months after implantation. The mean hearing threshold in a sound field before implantation was 64.4 dB HL and 37.4 dB HL after 12 months. Mean recognition score (WRS) measured before implantation was 13.5% and 87.6% after 12 months. Mean unaided speech reception threshold (SRT) measured before implantation was 4.36 dB SNR and − 1.95 dB SNR after 12 months. The outcomes of the audiological tests are comparable with the first published results, and the longer observation confirms stability.

The differences between the findings in the studies may be due to the size of the research groups and the results achieved by patients before implantation. The obtained performance is also comparable with the results of the first-generation implant. Generally, papers focus on audiological results and patient satisfaction, and in all published studies, the first-generation BCI 601 implant significantly improved hearing and speech understanding [8, 14, 25, 29–34].

Subjective evaluation of patients has confirmed improvements in speech understanding. Implantation had a positive impact on daily functioning and the patients’ quality of life. The implant improved communication even in difficult acoustic conditions [8, 30, 31]. Patients with the newer BCI 602 implant also perceive an improvement in hearing and speech understanding, which was confirmed in this study using the APHAB questionnaire. All subscales of the questionnaire showed a significant decrease in hearing problems after implantation. Additionally, in this study, the amount of change in the individual APHAB outcomes was gauged. A change of at least 22 points in the EC subscale was found in 45% of patients at the 1 month follow-up and in 52% of patients at the 6 month follow-up. On the BN subscale, it was 43% and 50%, and on the RV subscale 36% and 43%, respectively.

In the works published so far, both the first- and the second-generation implants have been shown to be safe solutions for the patient. In the literature, only ten major adverse events have so far been described for the BCI 601: one revision surgery due to headache [35], one implant removed because of local infection [33], one explanation due to device failure [36], one case of sudden loss of hearing benefit [35], two explanations’ due to lack of benefit (although these patients were outside the indication criteria) [34, 37], and four explanations after implantation into a radical cavity (three cases due to wound dehiscence) [34]. There are no reports of complications after the use of the new BCI 602. Sprinzlet al. [25] demonstrated that BCI 601 implantation has a low rate of minor adverse events (5.12%) or of revision surgery (0.85%). In our study, after 1 year of BCI 602 use, no revision surgery and no skin reactions were reported [24].

A significant advantage of the new implant is less time required for surgery. In this study, operation times for BCI 602 implantation ranged from 19 to 66 min (mean 28.3 min ± 9.4.) (*n* = 42). Implantation of the first-generation BCI 601 implant took longer, with times ranging from 28 to 57 min (mean 35.6 ± 8.1) (*n* = 10).

Comparing the obtained results with results of other bone-conduction devices, the BCI 602 implant compares favorably [31, 38–40]. The real mean functional gain after 12 months compared to before implantation was 27 dB HL. Hougaard et al. [41] reported that the functional gain in conductive or mixed hearing loss patients when using the BAHA Attract was 19.8 dB, which is lower than the improvement reported for the BB implant. Distinct advantages are good sound quality, a significant improvement in speech understanding, especially in noise, and visual aspects. The processor is small, it can be color-matched to the patient's hair, and a properly selected magnet does not cause undue pressure on the skin [42]. In addition, patients are given a remote control that allows them to quickly change the implant settings depending on the acoustic situation. Based on interviews and checking the data stored in the processor, the average time per day of using the implant was 9.6 ± 2.3 h. The processor is powered by batteries that patients replaced every 8.6 ± 1.6 days on average. The results obtained here are comparable with the results of the first published work on the BCI 602 [25]. From the patient’s perspective, the audiological outcomes, and subjective impressions indicate favorable benefits after implantation with either the BCI 601 or the BCI 602.

## Conclusion

The Bonebridge BCI 602 implant is an effective method of hearing rehabilitation in adult patients with conductive or mixed hearing loss. After 12 months, the BCI 602 provided significant improvements in objective and subjective auditory performance. The reduced thickness of the implant reduces surgical time and risk, especially in difficult anatomical conditions. The successful audiological results confirm the effectiveness of the implant, as do improvements in hearing and speech understanding and patient satisfaction. The hearing results after implantation of the BCI 602 were comparable to the published results of its predecessor, the BCI 601. Both the first- and second-generation implants confer significant benefits to the patient. Further observations of the results of patients with the new implant should be conducted to verify the stability of the results over several years and compare it with long-term follow-ups of the first-generation implant.

## References

[1] Hill-Feltham PR, Johansson ML, Hodgetts WE (2021). Hearing outcome measures for conductive and mixed hearing loss treatment in adults: a scoping review. Int J Audiol.

[2] Skarżyński H, Plichta Ł, Król B, Cywka KB, Skarżyński PH (2021). Implantation of the vibrant soundbridge in a case of bilateral malformation of the middle and external ear. Am J Case Rep.

[3] Cywka KB, Król B, Skarżyński PH (2021). Effectiveness of bone conduction hearing aids in young children with congenital aural atresia and microtia. Med Sci Monit.

[4] (199) Committee on Hearing and Equilibrium guidelines for the evaluation of results of treatment of conductive hearing loss. Am Acad Otolaryngol-Head Neck Surg Found Inc Otolaryngol Head Neck Surg 113(3):186–187. 10.1016/S0194-5998(95)70103-6 10.1016/S0194-5998(95)70103-6 7675477

[5] Phan NT, McKenzie JL, Huang L, Whitfield B, Chang A (2016). Diagnosis and management of hearing loss in elderly patients. Aust Fam Physician.

[6] Skarzynski PH, Ratuszniak A, Osinska K (2019). A comparative study of a novel adhesive bone conduction device and conventional treatment options for conductive hearing loss. Otol Neurotol.

[7] Magele A, Schoerg P, Stanek B, Gradl B, Sprinzl GM (2019). Active transcutaneous bone conduction hearing implants: systematic review and meta-analysis. PLoS One.

[8] Skarżyński PH, Ratuszniak A, Król B (2019). The Bonebridge in adults with mixed and conductive hearing loss: audiological and quality of life outcomes. AudiolNeurootol.

[9] Plontke SK, Götze G, Wenzel C, Rahne T, Mlynski R (2020). Implantation of a new active bone conduction hearing device with optimized geometry. HNO.

[10] Utrilla C, Gavilán J, García-Raya P, Calvino M, Lassaletta L (2021). MRI after Bonebridge implantation: a comparison of two implant generations. Eur Arch Otorhinolaryngol.

[11] Zernotti ME, Chiaraviglio MM, Mauricio SB, Tabernero PA, Zernotti M, Di Gregorio MF (2019). Audiological outcomes in patients with congenital aural atresia implanted with transcutaneous active bone conduction hearing implant. Int J Pediatr Otorhinolaryngol.

[12] Bravo-Torres S, Der-Mussa C, Fuentes-López E (2018). Active transcutaneous bone conduction implant: audiological results in paediatric patients with bilateral microtia associated with external auditory canal atresia. IntJ Audiol.

[13] Ngui LX, Tang IP (2018). Bonebridge transcutaneous bone conduction implant in children with congenital aural atresia: surgical and audiological outcomes. J Laryngol Otol.

[14] Ratuszniak A, Skarzynski PH, Gos E, Skarzynski H (2019). The Bonebridge implant in older children and adolescents with mixed or conductive hearing loss: audiological outcomes. Int J Pediatr Otorhinolaryngol.

[15] Weiss R, Leinung M, Baumann U, Weißgerber T, Rader T, Stöver T (2017). Improvement of speech perception in quiet and in noise without decreasing localization abilities with the bone conduction device Bonebridge. Eur Arch Otorhinolaryngol.

[16] Baumgartner WD, Hamzavi JS, Böheim K (2016). A new transcutaneous bone conduction hearing implant: short-term safety and efficacy in children. Otol sNeurotol.

[17] Canale A, Boggio V, Albera A (2019). A new bone conduction hearing aid to predict hearing outcome with an active implanted device. Eur Arch Otorhinolaryngol.

[18] Seiwerth I, Fröhlich L, Schilde S, Götze G, Plontke SK, Rahne T (2021). Clinical and functional results after implantation of the bonebridge, a semi-implantable, active transcutaneous bone conduction device, in children and adults. Eur Arch Otorhinolaryngol.

[19] Almuhawas FA, Dhanasingh AE, Mitrovic D (2020). Age as a factor of growth in mastoid thickness and skull width. Otol Neurotol.

[20] Andersen SAW, Bergman M, Keith JP (2021). Segmentation of temporal bone anatomy for patient-specific virtual reality simulation. Ann Otol Rhinol Laryngol.

[21] Nuesse T, Wiercinski B, Brand T, Holube I (2019). Measuring speech recognition with a matrix test using synthetic speech. Trends Hear.

[22] Löhler J, Gräbner F, Wollenberg B, Schlattmann P, Schönweiler R (2017). Sensitivity and specificity of the abbreviated profile of hearing aid benefit (APHAB). Eur Arch Otorhinolaryngol.

[23] Cox RM, Alexander GC (1995). The abbreviated profile of hearing aid benefit. Ear Hear.

[24] Alzhrani F (2019). Objective and subjective results of the Bonebridge transcutaneous active direct-drive bone conduction hearing implant. Saudi Med J.

[25] Sprinzl GM, Schoerg P, Ploder M, Edlinger SH, Magele A (2021). Surgical experience and early audiological outcomes with new active transcutaneous bone conduction implant. OtolNeurotol.

[26] Król B, Porowski M, Cywka KB, Skarżyńska MB, Skarżyński PH (2020). Mastoid obliteration with s53p4 bioactive glass can make bonebridge implantation feasible: a case report. Am J Case Rep.

[27] Król B, Cywka KB, Skarżyńska MB, Skarżyński PH (2021). Mastoid obliteration with S53P4 bioactive glass after canal wall down mastoidectomy: preliminary results. Am J Otolaryngol.

[28] Król B, Cywka KB, Skarżyńska MB, Skarżyński PH (2021). Implantation of the Bonebridge BCI 602 after mastoid obliteration with S53P4 bioactive glass: a safe method of treating difficult anatomical conditions-preliminary results. Life (Basel).

[29] Manrique M, Sanhueza I, Manrique R, de Abajo J (2014). A new bone conduction implant: surgical technique and results. Otol Neurotol.

[30] Eberhard KE, Olsen SØ, Miyazaki H, Bille M, Caye-Thomasen P (2016). Objective and subjective outcome of a new transcutaneous bone conduction hearing device: halfyear follow-up of the first 12 nordic implantations. OtolNeurotol.

[31] Schmerber S, Deguine O, Marx M (2017). Safety and effectiveness of the Bonebridge transcutaneous active direct-drive bone-conduction hearing implant at 1-year device use. Eur Arch Otorhinolaryngol.

[32] Bianchin G, Bonali M, Russo M, Tribi L (2015). Active bone conduction system: outcomes with the Bonebridge transcutaneous device. ORL J Otorhinolaryngol Relat Spec.

[33] Fan X, Yang T, Niu X, Wang Y, Fan Y, Chen X (2019). Long-term outcomes of bone conduction hearing implants in patients with bilateral microtia-atresia. Otol Neurotol.

[34] Brkic FF, Riss D, Scheuba K (2019). Medical, technical and audiological outcomes of hearing rehabilitation with the bonebridge transcutaneous bone-conduction implant: a single-center experience. J Clin Med.

[35] Rader T, Stöver T, Lenarz T (2018). Retrospective analysis of hearing-impaired adult patients treated with an active transcutaneous bone conduction implant. Otol Neurotol.

[36] Wimmer W, von Werdt M, Mantokoudis G, Anschuetz L, Kompis M, Caversaccio M (2019). Outcome prediction for Bonebridge candidates based on audiological indication criteria. Auris Nasus Larynx.

[37] Riss D, Arnoldner C, Baumgartner WD (2014). Indication criteria and outcomes with the Bonebridge transcutaneous bone-conduction implant. Laryngoscope.

[38] Gerdes T, Salcher RB, Schwab B, Lenarz T, Maier H (2016). Comparison of audiological results between a transcutaneous and a percutaneous bone conduction instrument in conductive hearing loss. Otol Neurotol.

[39] Zernotti ME, Di Gregorio MF, Galeazzi P, Tabernero P (2016). Comparative outcomes of active and passive hearing devices by transcutaneous bone conduction. Acta Otolaryngol.

[40] Goycoolea M, Ribalta G, Tocornal F (2020). Clinical performance of the OsiaTM system, a new active osseointegrated implant system. Results from a prospective clinical investigation. Acta Otolaryngol.

[41] Hougaard DD, Boldsen SK, Jensen AM, Hansen S, Thomassen PC (2017). A multicenter study on objective and subjective benefits with a transcutaneous bone-anchored hearing aid device: first Nordic results. Eur Arch Otorhinolaryngol.

[42] Billinger-Finke M, Bräcker T, Weber A, Amann E, Anderson I, Batsoulis C (2020). Development and validation of the audio processor satisfaction questionnaire (APSQ) for hearing implant users. Int J Audiol.

